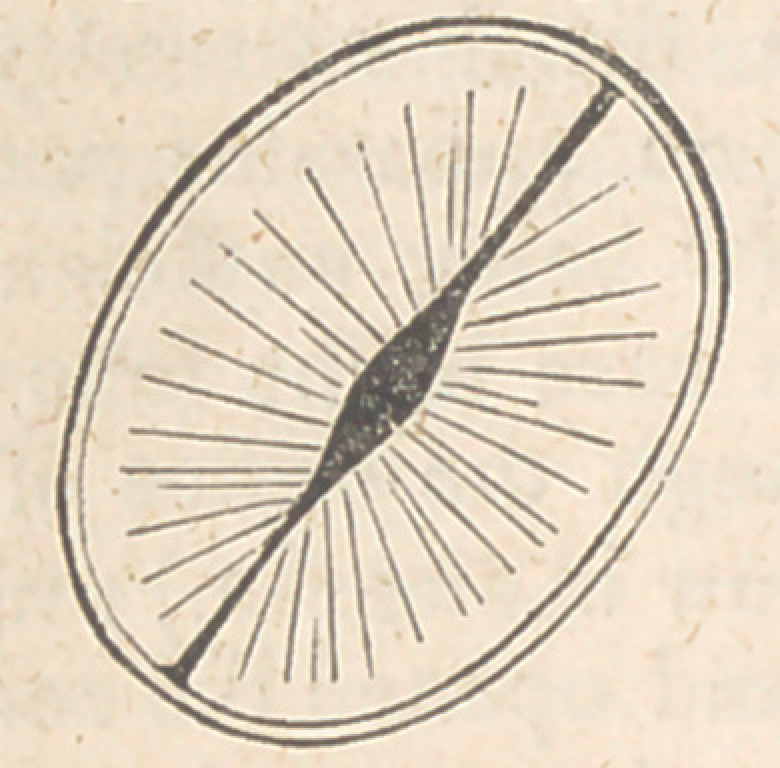# Experimental Inquiry into the Causes of the Sounds of the Heart, Confirmatory of the Views of Dr. Billing

**Published:** 1850-01

**Authors:** Harris Carr Brakyn

**Affiliations:** Trinity College, Dublin


### RECORD OF MEDICAL SCIENCE.

### ANATOMY AND PHYSIOLOGY.

### *Experimental Inquiry into the Causes of the Sounds of the Heart, Confirmatory of the Views of Dr. Billing.*

By Harris C. Brakyn, Esq., Trinity College, Dublin.

*Dublin, Mercer's Hospital, William street, July* 20, 1849.

To Archibald Billing, M. D.

Sir,—The following communication is addressed to you by a very unpretending student in medicine, trusting the liberty of so doing will be excused, in consideration of its being offered in a spirit of perfectly respectful well meaning, even should the task prove superfluous.

I enclose you a very rude diagram of an experiment upon a heart, designed as an illustrative proof of your doctrine of the sounds of that organ, the history of which is as follows:—Having had the good fortune, last year, to become possessed of a copy of your work on "The Principles of Medicine," I became, through it, first acquainted with an intelligible theory of the heart-sounds, with which acquisition I was intensely pleased, both on account of its beautiful simplicity, and the pleasure of surmounting an obstacle which had hitherto been to me a *pons asinorum.* As to the very great pleasure and profit I derived from the study of the rest of the book, it will be at present unnecessary to speak further.

To my surprise, Sir, however, I found that the doctrines which you had so courageously, and, in my opinion, successfully exploded, were still believed and taught, with but one or two exceptions, by the most distinguished practitioners and professors in Dublin. The knowledge of this fact made me, of course, but the more anxious to see sound doctrine promulgated: and accordingly, on every available opportunity, I took occasion to produce your book, and discuss your opinions, but I am sorry to say, by no means with the success I could desire. I found that the amount of self-satisfied ignorance, indifference, or stupidity, which was such a very ordinary accompaniment to laborious study and reputed acquirement, was so great, that notwithstanding the weight your name carried, I could seldom obtain more than the liberty of holding my own opinions, under the protection of so eminent an authority.

This being an exceedingly unsatisfactory state of things, it occurred to me as very desirable, and not impossible, to prove the truth of your doctrines by the irrefragable authority of a *legitimate experimentum crucis.* With this hope, during March and April last, I commenced a series of experiments on ox-hearts, which I had finally the extreme gratification of finding succeed to my utmost wishes. After several trials in private to the entire conviction of some intelligent friends, I

was invited by Professor Fergusson, whose clinique I was attending, to exhibit the experiment to the class in Sir Patrick Dunn's Hospital.

The result was triumphant : the *double* sound of the valves, when thrown by alternating currents of air into tympanic tension, could be heard all over the room. Dr. Fergusson, who had long held a doctrine coinciding with yours, was much pleased, and kind enough to honor the experiment with very liberal commendation.

Some time after, my private teacher, Mr. T. Ledwick, of York-street, requested me to lay the matter before Dr. Lees, of the Meath Hospital, who did not agree with your views. He seemed much surprised, and very unwilling to admit the evidence before him, until after I had satisfied him by repeated examinations, alterations, and modifications of the experiment; when, with much candor, he admitted that he was unable to offer any further opposition to the opinion advocated by the experiment. Dr. Lees further very kindly introduced the subject to the notice of his distinguished colleague, Dr. Stokes (author of "Diseases of the Chest,") who, after a private examination, appointed a morning for me to attend him at lecture, when he delivered a well digested *resumé* of the various doctrines of the cardiac sounds—during the course of which I had the very gratifying honor of being presented by the Doctor to his class, with the request to exhibit and explain the experiment, which I did, to the best of my ability, as an illustration suggested by Dr. Billing's writings, in support of his theory.

The success was perfect, and seemed to give Dr. Stokes very great satisfaction, who finally pronounced the experiment *conclusive*. This public recognition of a much cherished opinion by so distinguished an authority, gave me extreme satisfaction, in which, I trust I do not presume too much in hoping that Dr. Billing may somewhat participate.

I have still preserved in my possession the apparatus used on the last mentioned occasion, which consists of an ox-heart carefully dissected from the animal, so as to avoid injury to any of the appendages. To this (as displayed in diagram) I attached an apparatus consisting of a flanged tube, *a*, attached to the middle of left ventricle, and piercing its wall, introduced through the auriculo-ventricular opening, to which was screwed externally another tube, *b*, with a flange also, so as to grasp the wall of ventricle all round the tube, and render the junction air-tight; to the outer tube a bladder is tied. A free communication is thus established between the bladder externally and the cavity of the ventricle within. To the left auricle, a similar apparatus, but without flanges, was then attached by one trunk of the pulmonary veins, the rest being tied. Then having tied all the offsets of the aorta, I tied a tube and bladder to its abdominal extremity; to the distal end of this a small stop-cock was then tied, into which a brass pipe fastened to the end of an india-rubber one, can be wedged; the other extremity of the caoutchouc pipe is finally attached to the distal extremity of the auricular bladder. There is thus completed an apparatus permitting a mimic circulation through the left heart, (*it* being sufficient for illustration,) which may be conducted with great ease in the following mode. Let the system be inflated with air through the orifice of the elastic tube next to the stop-cock (marked by an arrow in diagram); when, having wedged back the stop-cock into the pipe and opened the cock, a rhythmical circulation may be carried on by alternating manual pressure applied to each of the three bladders in succession (without removing *any* of the *three* hands applied)—thus *representing* the successive contractions of auricle, ventricle, and aorta, with the natural attempts at regurgitation, which close both sets of valves in succession. Hereby a complete imitation of the normal sounds may be produced on either a very magnified or diminished scale, according to the force used in propelling the air. These sounds being produced without any muscular contraction, or rush of blood, &c., must evidently be *valvular*, which can be further demonstrated by removing part of the apparatus, (the auricular,) so as to show the mitral valves in action synchronously with the first sound; or by introducing a wire cage, prevent them closing on regurgitation, when no sound follows: above all, the first sound is as perfect as the second, the valvular origin of which is, I believe, undisputed. In fine, the illustration, though conducted with air, ought to be conclusive, inasmuch as a suddenly strained membrane, which gives a tympanitic sound in air, will do the same in water also, as I have tried, but not so loudly.

Dr. Stokes, also, in subsequent conversations with me, mentioned a fact which he had noticed several times in typhus fever, and which he seemed to think likely to favor your doctrine—viz., that patients, after the fever had progressed some time, and great debility had supervened, ceased to have any sound of the heart, though circulation continued. Sometimes, however, the second continued, the first being absent ; but it was to be remarked in these cases, that the impulse of the heart was quite absent. This, no doubt, Sir, the muscular theorists would endeavor to appropriate, as well as the supporters of your opinions. I imagine, however, that this additional fact would not be so easy of digestion to the former gentlemen, which is, that as the patients improved, impulse was found to return, often with considerable energy, but unaccompanied for some days by any sounds, or, in some instances, by the second only. This state of things can be also imitated most accurately by the bladder apparatus.

I also endeavored to try the experiment with water, but did not succeed, for want of a proper hydraulic apparatus of adequate power, which would have been too expensive and laborious an undertaking for me to have ventured on. I should have mentioned before, a fact of some interest in connexion with the character of sound produced by the valves—namely, that when the mitrals were made to act whilst exposed to view, the sound could be produced by the sudden straining of the valves, when the ventricular bladder was pressed, even though the valves had been previously in contact, or with a very minute orifice existing by their partial patency, such as in the accompanying diagram.

This fact would seem to me to disprove the statements made in several works of eminence, that the second sound is produced by a click of the sigmoid valves ; the first being regarded by such authors as muscular, whilst the above fact must, I think, prove your assertion of both being "tympanitic."

In apologizing for the great length of the present communication, I would beg to state that I had intended reserving it for a personal interview with Dr. Billing, in London, with which I hoped to have been favored during the ensuing month; but as certain circumstances have lately occurred, which I fear may possibly compel me to defer that visit indefinitely, I have thought it, Sir, more judicious to acquaint you with the foregoing facts, as, should they happen to afford you any satisfaction, or not to have been anticipated, the sooner you were possessed of them the better; for I considered it would have been both imprudent and indecorous in me to have brought the matter any further into notice than I have mentioned, which was, indeed, all but unavoidable.

In conclusion, I have, Sir, but to apologize for the liberty I have taken with your name and opinions; and venturing to hope that my humble efforts may not be altogether unacceptable, beg to place them, with much respect and esteem, entirely at your service.

I am, Sir, your very obedient servant,

HARRIS CARR BRAKYN, T. C. D.

## Figures and Tables

**Figure f1:**
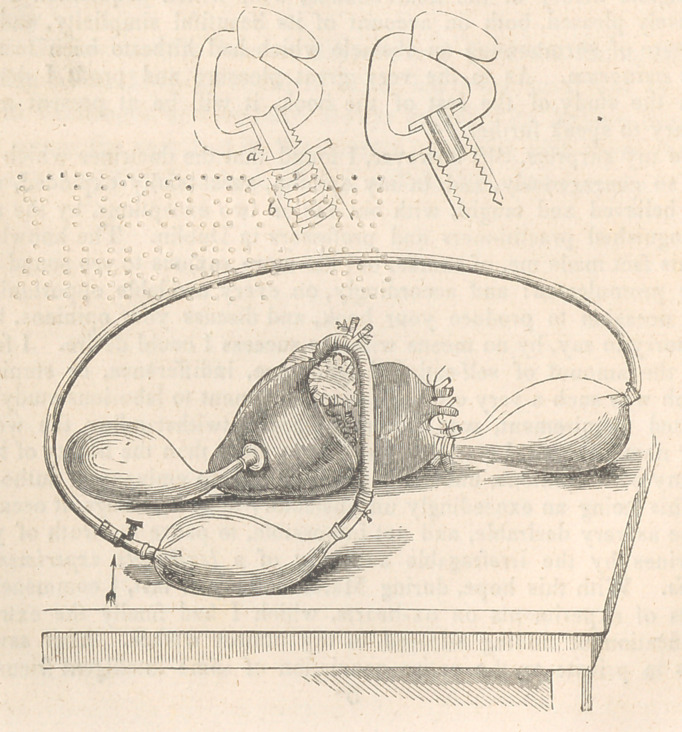


**Figure f2:**